# Delayed Treatment of Traumatized Primary Teeth with Distinct Pulp Response: Follow-Up until Permanent Successors Eruption

**DOI:** 10.1155/2017/3157453

**Published:** 2017-03-29

**Authors:** Gabriela Cristina de Oliveira, Juliana Calistro da Silva, Franciny Querobim Ionta, Catarina Ribeiro Barros de Alencar, Priscilla Santana Pinto Gonçalves, Thaís Marchini de Oliveira, Thiago Cruvinel, Daniela Rios

**Affiliations:** ^1^Department of Pediatric Dentistry, Orthodontics, and Public Health, Bauru Dental School, University of São Paulo, Alameda Dr. Octávio Pinheiro Brisolla 9-75, P.O. Box 73, 17012-101 Bauru, SP, Brazil; ^2^Department of Dentistry, Paraiba State University, Av. Cel. Pedro Targino, s/n, Centro, 58233-000 Araruna, PB, Brazil

## Abstract

Complicated crown fracture and crown-root fracture with pulp involvement expose dental pulp to the oral environment. The pulp outcome is often unpredictable because the patient and injury which are related to variables can influence the treatment of choice and the prognosis of the case. This report presents the case of a 4-year-old boy with complicated crown fracture with pulp polyp in the primary right maxillary central incisor** (51)** and crown-root fracture with pulp involvement in the primary left maxillary central incisor** (61)**, which was treated only 3 months after the tooth injuries. The treatment of choice was extraction of tooth** (61)** due to a periapical lesion with disruption of the dental follicle of the permanent successor and pulpotomy (MTA) of the tooth** (51)**, because the pulp presented signs of vitality. At the follow-up visits, no clinical, symptomalogical, and radiographic changes were observed until the primary tooth's exfoliation. However, at 3-year follow-up, the permanent successors showed hypocalcification and the position of the permanent right maxillary central incisors** (11)** was altered. Besides the conservative and adequate delayed treatment, the sequelae on the permanent successors could not be avoided.

## 1. Introduction

Traumatic injuries to the primary teeth are the second most frequent cause of consultation in pediatric dental practice. In preschool children, the periodontal ligament is very elastic and the alveolar process is characterized by large bone marrow spaces; therefore the teeth are less firmly held in place. As a result, in the event of a slight traumatic injury, damage to the supporting tissue is more common than to the hard tissue of the tooth [1]. Besides being less frequent, the traumatic crown injuries are considered a public dental health problem due to its costs and treatment, which may persist for the rest of the patient's infancy [2]. Particularly among Brazilian preschool children, a recent investigation has shown that the prevalence of traumatic crown injuries increased in the last 10 years [2]. The most commonly affected primary teeth are the upper central incisors and more than one tooth can be lesioned [1].

Complicated crown fracture and crown-root fracture with pulp involvement expose dental pulp to the oral environment. The time elapsed between accident and treatment is an important factor to consider before deciding the therapeutic approach for the traumatically exposed pulp [3]. The prompt treatment of pulp is important to minimize bacterial invasion and ensure pulpal healing, preventing necrosis of the primary tooth. However, the dental pulp can evolve to a hyperplasia and resists to necrosis, showing resistance and reactivity against bacterial infection [4]. In addition, the inadequate treatment and the impact resulting from the traumatic injury can cause complications, ranging from minor negative effects to significant consequences for either the injured primary tooth or its permanent successor [5–8]. The present case reports distinct responses of traumatically exposed pulp of primary central incisors, the conservative delayed treatment of the pulp polyp, and the consequences of the trauma to the permanent successor teeth.

## 2. Description of Case

A 4-year-old healthy boy presented with his mother to the Clinic of Pediatric Dentistry of Bauru Dental School, University of São Paulo (Brazil). The chief complaint was the aesthetic impairment of the maxillary central incisors.

### 2.1. Case History

A detailed history revealed that the boy fell to the floor and had a dental trauma 3 months before. At that time, the child was immediately conducted to an emergency dental care center but no intervention was performed due to the uncooperative child behavior. The dentist explained to the responsible for the child that there was no need for treatment because the teeth would exfoliate soon. In addition, the parents were not told to seek for specialized dental care.

### 2.2. Intraoral Findings

**Figure 1:**
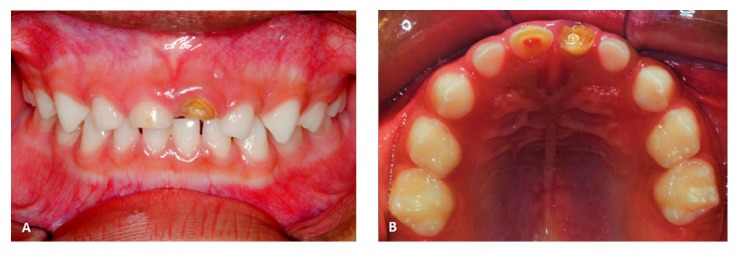
(A) Facial view of traumatized teeth, enamel-dentin-pulp fracture of** (51),** and crown-root fracture with pulp involvement (fistula) of** (61)**. (B) Occlusal view of** (51)** and** (61)** note the presence of pulp polyp** (61)**.

Intraoral examination showed no caries lesions, enamel-dentin-pulp fracture with pulp polyp in the primary right maxillary central incisor** (51),** and crown-root fracture with pulp involvement in the primary left maxillary central incisor** (61)** (Figures 1(A) and 1(B)). A fistula was observed in the apical region of the root of** (61)** (Figure 1(B)). The patient was asymptomatic.

### 2.3. Radiographic Examination

The initial periapical radiography was performed using the occlusal modified technique with a number 2 radiographic film in an occlusal position. The child kept the radiographic film in the correct position by biting. The cone of the X-ray apparatus was positioned at the top of the patient's nose angled between +35° and +45°. The follow-up periapical radiography was performed using the bisected angle technique with a number 0 radiographic film and dental radiographic positioner.

**Figure 2:**
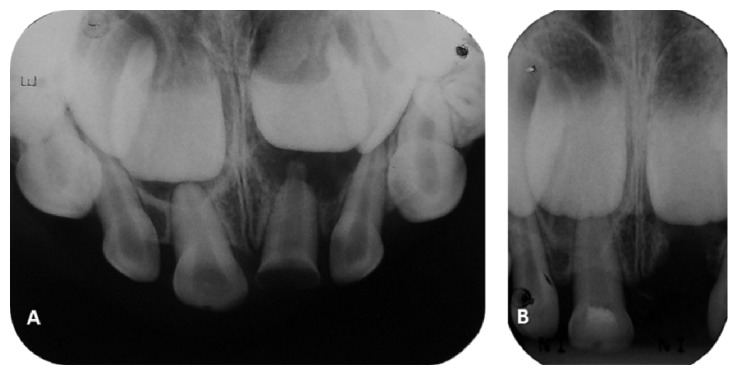
(A) The initial periapical radiography showing no periapical alterations in** (51)** and lesion in** (61)** with disruption of the dental follicle of the permanent left maxillary central incisor** (21)**. (B) The periapical radiography after the pulpotomy of tooth** (51)** and the extraction of** (61)**.

Periapical radiograph examination revealed periapical lesion with disruption of the dental follicle of the permanent left maxillary central incisor** (21)** and height differences between permanent dental germs (Figure 2(A)). No periapical alterations were observed in** (51)** (Figure 2(A)).

### 2.4. Treatment

The informed consent was obtained from his parents and the child was subject to the treatment. The extraction of tooth** (61)** was performed. Considering the presence of pulp polyp on tooth** (51)**, an endodontic intervention was proposed. The coronal access was done with high speed diamond bur under a rubber dam and local anesthesia. After coronal pulp amputation a bright red hemorrhage was observed. Continuous rinsing of the amputated pulp with saline achieved hemostasis without blood clot formation within 4 minutes. The treatment of choice was coronal pulpotomy and the pulp chamber was dressed with MTA (mineral trioxide aggregate) (Angelus®, Londrina, PR, Brazil), followed by a base/liner of calcium hydroxide cement and restoration with composite resin (Figure 2(B)).

**Figure 3:**
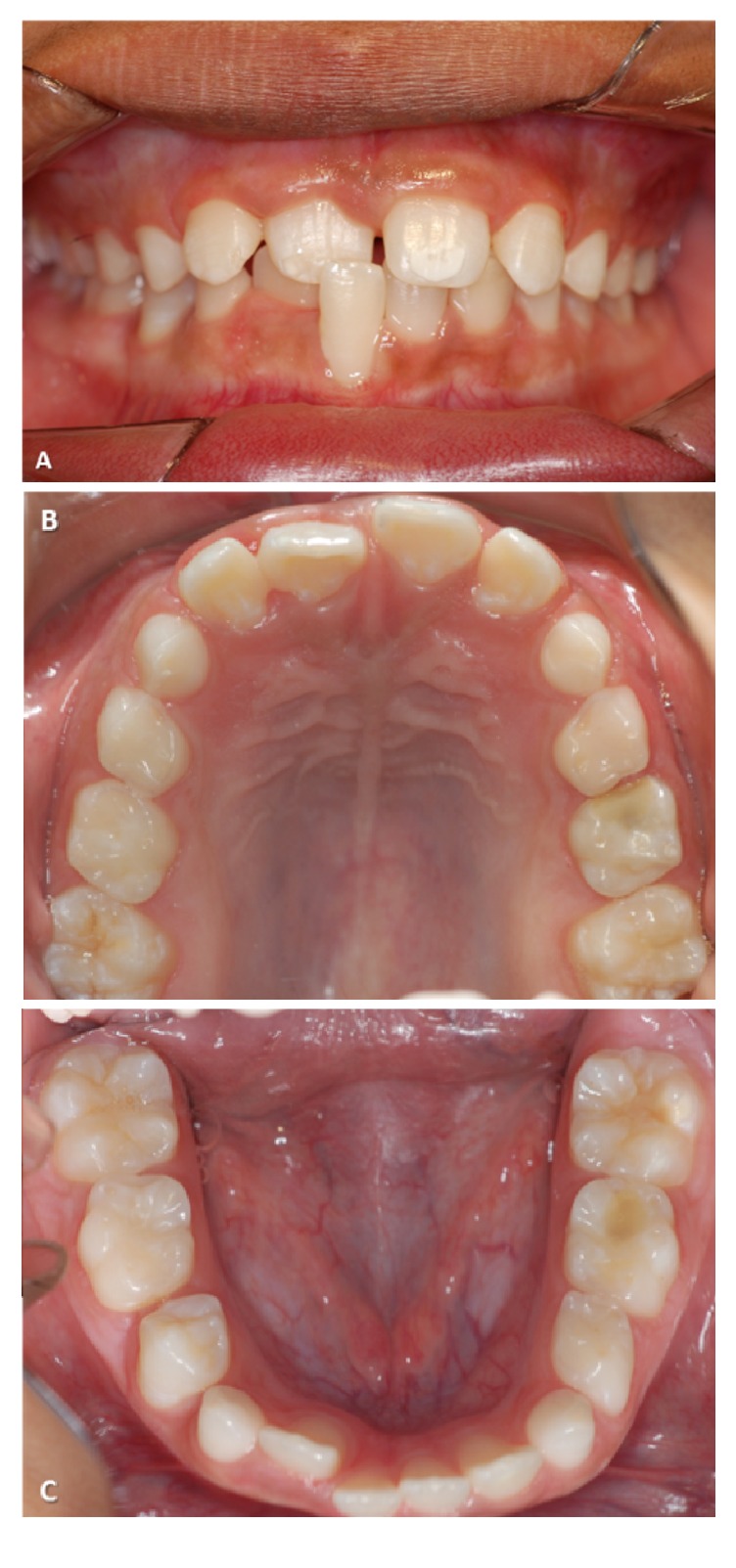
Facial view of permanent teeth, note the erupted position change of the tooth** (11)** in the dental arch and the tooth** (11)** with enamel hypocalcification.

Scheduled follow-ups, after 1 month, after 3 months, and then every 6 months, were made and no clinical, symptomalogical, and radiographic changes were observed. After a month, as the child presented good oral hygiene, a prosthetic device acrylic palatal device with artificial primary central incisor in the region tooth** (61)** for aesthetic purposes was prepared. The tooth** (51)** remained in mouth until the eruption of the permanent successor. At 3-year follow-up the permanent right and left maxillary central incisors** (11** and** 21)** showed hypocalcification (Figures 3(A), 3(B), and 3(C)), being more severe in tooth** (21)**. Tooth** (11)** showed position alteration (Figure 3(a)). The right upper first permanent molar (Figure 3(B)) and the left lower first permanent molar (Figure 3(C)) also showed hypocalcification.

## 3. Discussion

In the present case, the enamel-dentin fracture occurred on the palatal area of the tooth. The pulp was exposed to thermal changes and mastication for 3 months without sensitive tooth or pain. Despite the oral insults the pulp resisted to necrosis [9], showing a reddish mucosa-like structure with the same size as the crown, characterizing a pulp polyp [4]. On the other hand, the same accident resulted in crown-root fracture with pulp involvement of the adjacent tooth, which progressed to pulp necrosis. Probably the intensity of the trauma was higher for the tooth** (61)** resulting in no pulp healing ability [1]. The occurrence of pulp polyp in traumatized primary teeth is not common and there is a greater chance to occur in children under 2 years of age [4]. Previous study showed that delayed treatment was negatively associated with pulp polyp occurrence, probably because polyp can be interpreted by parents as a condition that requires attention [4]. However, in the present case, the polyp occurred in the palatal area; this was not the chief complaint, justifying the delay in seeking treatment.

The treatment for the pulp polyp was cervical pulpotomy because following coronal pulp amputation, the bleeding appeared normal in color, no excessive bleeding was present, and good hemostasis was achieved [10]. The material used for the pulpotomy was MTA due to its biocompatibility, ability to induce the formation of tertiary dentine, and promotion of a proper sealing [11]. The influence of MTA pulpotomies in primary teeth upon eruption and calcification of permanent successors was recorded after exfoliation of the treated molars [12]. The results showed no alterations in color, mineralization, structure or position, and no alterations in the timing of eruption [12]. However, in the present case, the permanent successor of the primary tooth with pulpotomy showed alteration on mineralization and position. The etiology of permanent central incisors hypomineralization should be analyzed with caution. Since two first permanent molars also showed hypomineralization, the incisor could be part of molar incisor hypomineralization (MIH) [13]. MIH is a common developmental dental defect characterized by demarcated, qualitative defects of enamel affecting one or more first permanent molars with or without incisor involvement [14]. Besides being from a systemic origin, there is no consensus regarding the etiology of MIH [13, 15]. The condition has been described as a genetic component related to disturbances in the maturation stages of enamel [15] or associated with a variety of etiological factors [13].

Another etiological cause for the central incisors alteration could be the impact of the own trauma [1, 16] or the delay on treatment. The dentist should carry out as soon as possible the correct diagnosis and treatment of the dental trauma, due to the influence of time on case prognosis. In this case report, the patient did not receive the ideal treatment immediately after the trauma, leading to periapical injury on the left primary incisor. Pulp necrosis and the disruption of the dental follicle of the permanent can be considered aggressors for the permanent tooth development. This could have influence on the occurrence of enamel hypomineralization of the left permanent central incisor. In addition, the degree of complication for the permanent successor depends mainly on the development stage of the permanent tooth and the intensity of the trauma [6, 7]. Previous study shows that 53% of the successor tooth was affected after trauma to primary tooth in children between 3 and 4 years old [17]. Therefore, type and high intensity of the trauma might also be the reason for the hypomineralization of the left permanent central incisor in the present case.

In conclusion, according to the type and intensity of the trauma, distinct pulp response can be found in the same patient. In additional, the delayed treatment must consider the pulp condition, being conservative when possible and the correct treatment of the primary traumatized teeth is not guarantee of no sequelae on the permanent successors.
